# Nephrotic syndrome associated with Guillain–Barré syndrome and Sjögren syndrome: A case report and literature review

**DOI:** 10.1097/MD.0000000000043824

**Published:** 2025-08-15

**Authors:** Ping Guo, Ping Zhang, Jia Wei Zhao, Amanda Y. Wang, Wei Wang

**Affiliations:** a School of Medicine, University of Electronic Science and Technology of China, Chengdu, China; b Renal Department and Nephrology Institute, Sichuan Provincial People’s Hospital, School of Medicine, University of Electronic Science and Technology of China, Chengdu, China; c Faculty of Health Sciences & Medicine, Bond University, Robina, QLD, Australia; d Faculty of Medicine and Health Sciences, Macquarie University, Macquarie Park, NSW, Australia; e Renal and Metabolic Division, The George Institute for Global Health, UNSW Australia, Sydney, NSW, Australia; f Department of Renal Medicine, Concord Repatriation General Hospital, Concord Clinical School, University of Sydney, Camperdown, NSW, Australia.

**Keywords:** Guillain–Barré syndrome, minimal change disease, nephrotic syndrome, Sjögren syndrome

## Abstract

**Rationale:**

The coexistence of Guillain–Barré syndrome (GBS) and minimal change disease (MCD) is extremely rare. GBS is an autoimmune-mediated peripheral neuropathy that can occasionally be associated with renal complications such as nephrotic syndrome (NS). This case discusses a patient diagnosed with both GBS and MCD, as well as Sjögren syndrome kidney injury, focusing on the potential pathogenesis of these conditions and the role of autoantibodies in their development and treatment outcomes.

**Patient concerns:**

A young female patient presented with progressively worsening muscle weakness, sensory abnormalities, and edema. Further investigations revealed NS, characterized by proteinuria and hypoalbuminemia. While the neurological symptoms improved initially, the renal manifestations persisted, raising concerns about ongoing kidney damage. In addition, the patient was also found to have Sjögren syndrome kidney injury, along with positive perinuclear antineutrophil cytoplasmic antibody and antinuclear antibody, suggesting an autoimmune-mediated process contributing to the co-occurrence of these conditions.

**Diagnoses:**

GBS was diagnosed based on characteristic ascending paralysis and demyelination, as evidenced by nerve conduction studies. The diagnosis of MCD was supported by the patient’s clinical presentation of NS and kidney biopsy findings. The presence of clinical features such as dry mouth and dry eyes, coupled with positive anti-SSA/Ro52 and anti–Sjögren syndrome antigen B antibodies, pointed to Sjögren syndrome. Kidney biopsy results strongly suggested that kidney damage was likely due to Sjögren syndrome.

**Interventions:**

The patient was started on immunosuppressive therapy, including prednisone and cyclophosphamide, to address both the autoimmune neuropathy and renal issues. In addition, intravenous immunoglobulin was administered to treat the GBS. Supportive therapies, such as diuretics and albumin infusions, were used to manage edema and protein loss associated with NS.

**Outcomes:**

The patient showed significant improvement in neurological symptoms, including enhanced muscle strength and reduced sensory deficits. Proteinuria decreased, and renal function gradually stabilized.

**Lessons:**

This case illustrates the rare coexistence of GBS, MCD, and Sjögren syndrome kidney injury in a single patient. Autoimmune markers played a pivotal role in the pathogenesis of these diseases. Immunosuppressive therapy and intravenous immunoglobulin were essential in treating both neurological and renal complications. Further research is needed to deepen our understanding of the overlap of autoimmune diseases and to optimize treatment strategies for such complex cases.

## 1. Introduction

Minimal change disease(MCD) is the third most prevalent cause of idiopathic nephrotic syndrome (NS) in adults.^[1]^ Guillain–Barré syndrome (GBS) is an autoimmune-mediated polyradiculoneuropathy.^[2]^ Despite the paucity of relevant case reports of comorbid NS and GBS, the association of GBS with NS has been recognized as early as >100 years ago. Of the pathological types of NS combined with GBS, which included membranous nephropathy (MN),^[3]^ focal segmental glomerulosclerosis,^[4]^ MCD,^[5,6]^ MN was the most commonly reported. NS presenting as minimal change disease is rare in patients with GBS. Sjögren syndrome (SS) is a chronic autoimmune disease with rare renal involvement. Tubular interstitial nephritis (TIN) is a typical renal complication of SS, while glomerular injury, especially MCD, has only been rarely reported.^[7]^ We now described a case of NS, where the pathological type was MCD, comorbid with GBS and SS related renal injury followed by a literature review and discussion of the characteristics, pathogenesis, and combination therapy of these diseases.

## 2. Case report

A 31-year-old female patient initially presented with a fever of unknown origin and fatigue. She was initially treated with antipyretic drugs (specifics unknown) and the symptoms subsided. Approximately a couple of months, she began to experience persistent numbness of the left lower limb, associated with progressive weakness of both lower limbs, and twitching of both lower extremities twice, each lasting about 10 seconds with spontaneous relief. She was admitted to our hospital for further management. On examination, the patient was conscious, fully oriented, and in a normal mental state. Her temperature was 36.7 °C, heart rate was 125 beats per minute, respiration rate was 20 breaths per minute and, blood pressure was 93/60 mm Hg. Neurological examination revealed that the patient had no bulbar palsy or flaccid paraplegia, muscle strength of both lower limbs was 4-/5, and tendon reflexes were normal. Her past medical was not previous renal, neurological or rheumatic disease, family history of drug, heavy metal, toxin or disease exposure history, and recent vaccination history.

The laboratory investigations showed nephrotic-range proteinuria, with 24-hour urinary protein quantification of 6.544 g (peaking at 9.5 g) upon admission, serum creatinine of 37.7 μmol/L, and an estimated glomerular filtration rate of 136.33 mL/min. Complement 3 and complement 4 were within normal ranges. White blood cell counts were 5.320 * 10^9^/L with lymphocytes of 0.926 * 10^9^/L and the lymphocyte rate was 17.4%. During the disease course, the patient developed hypoalbuminemia (lowest serum albumin 19.5 g/L), persistent hypokalemia (lowest serum potassium 2.95 g/L), and transient urinary pH elevation to 7.5. Initial autoimmune serology showed anti-double-stranded DNA (‐), antinuclear antibody (ANA) positivity (titer 1:1000, granular pattern), anti-Sjögren syndrome antigen A (anti-SSA)/Ro52 (+++), anti-Sjögren syndrome antigen B (anti-SSB) (++), perinuclear anti-neutrophil cytoplasmic antibody (p-ANCA, +), and anti-mitochondrial antibody type M2 (AMA-M2, +). Ultrasound examination showed increased aortic renal blood flow resistance (left Vmax 78.2 cm/s, right Vmax 70.1 cm/s) and enlarged kidneys (left 12.3 × 4.2 cm, right 12.5 × 4.3 cm).

Electromyography showed that the peripheral nerves were damaged, especially in the lower extremities, and the symmetrical demyelination of the lower extremities was dominant. Brain MRI showed scattered small ischemic lesions in the left sub-frontal lobe area. A lumbar puncture demonstrated a quantitative cerebrospinal fluid protein of 1.08 g/L, a cerebrospinal fluid glucose of 5.10 mmol/L, a normal intracranial level and cerebrospinal fluid cell count. From the above findings, we diagnosed her with GBS according to the National Institute of Neurological Disorders and Stroke and Brighton clinical criteria.

**Figure 1.**
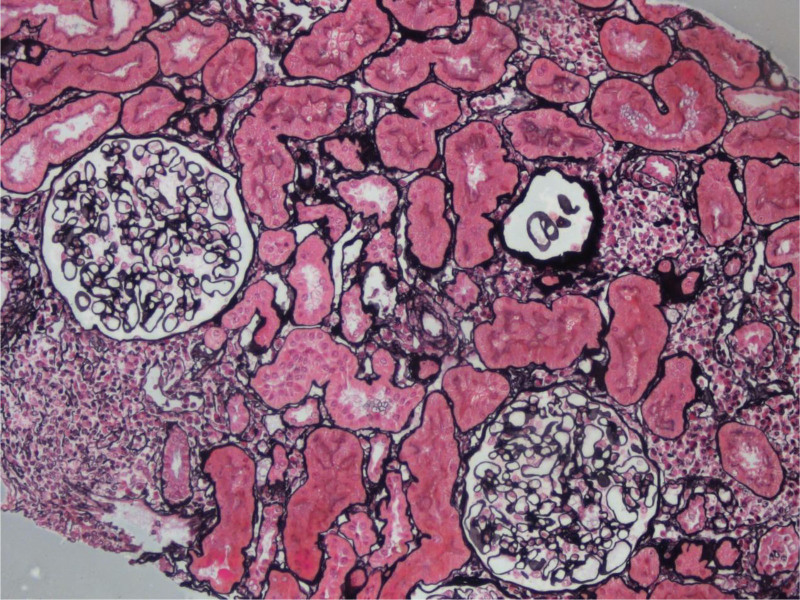
The renal tubules showed epithelial cell vacuoles and granular degeneration, small focal tubular atrophy and mild interstitial fibrosis (<5%), and renal interstitial multifocal lymphoid. Monocytes and more plasma cells infiltrates were seen (20–25%), and no obvious lesions were seen in the arteriole. Periodic acid-silver methenamine (PASM), magnification 100.

**Figure 2.**
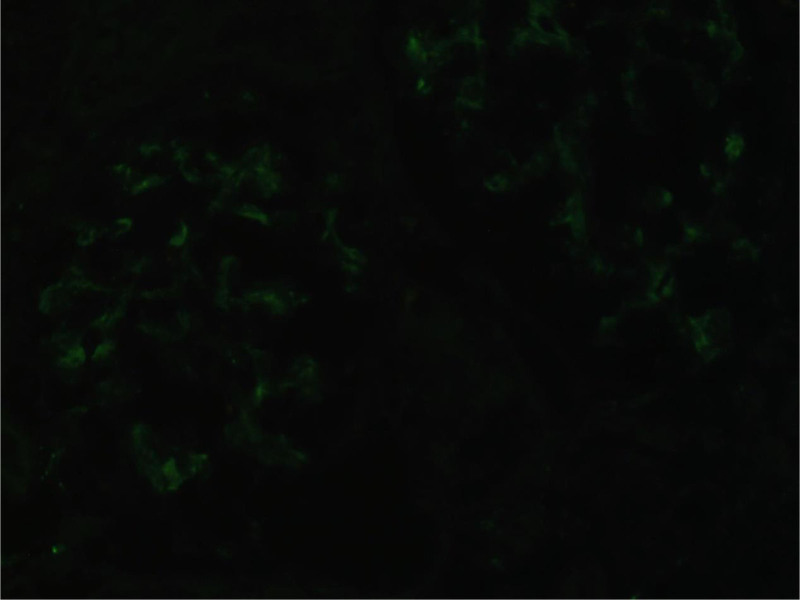
Immunofluorescence staining. Three glomeruli were shown here, IgG‐ and IgM+ mesangial block-like deposits were seen. IgA, C3, C1q, FRA, Alb, K, λ were all negative.

**Figure 3.**
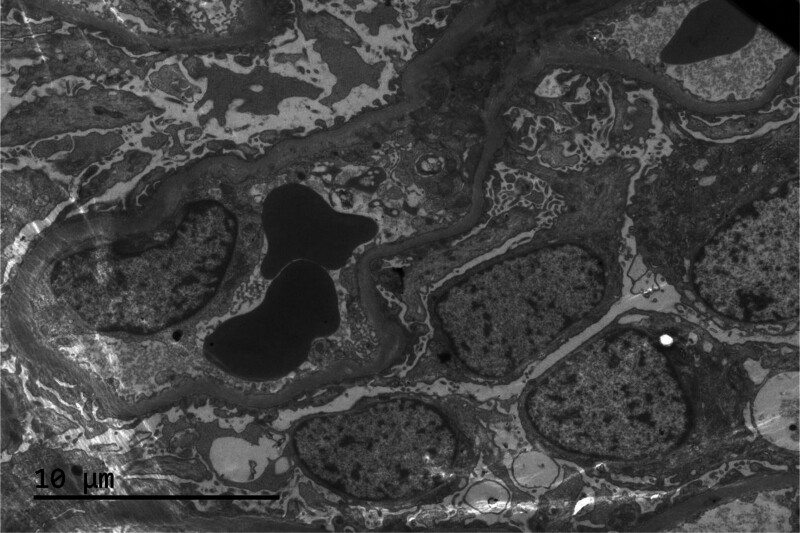
Electron microscopy, magnification 1000. Segmental mild hyperplasia of mesangial cells and matrix was seen, no obvious lesions were found in the basement membrane, the opening of the capillary lumen was basically good, most of the epithelial cell foot processes were fused, and no electron-dense deposits were seen.

Subsequently the patient underwent a percutaneous renal biopsy to diagnose renal disease histologically. Under the light microscope, the glomeruli showed only minimal lesions. The renal tubules showed epithelial cell vacuoles and granular degeneration, small focal tubular atrophy and mild interstitial fibrosis (<5%), and renal interstitial multifocal lymphoid. Monocytes and more plasma cells infiltrates were seen (20–25%), and no obvious lesions were seen in the arterioles (Fig. 1). Under immunofluorescence, IgG‐ and IgM+ mesangial block-like deposits were seen (Fig. 2). Under the electron microscope, segmental mild hyperplasia of mesangial cells and matrix was seen. No obvious lesions were found in the basement membrane. The opening of the capillary lumen was patent, most of the epithelial cell foot processes were fused, and no electron-dense deposits were seen (Fig. 3). Notably, despite severe proteinuria, the patient lacked hallmark NS manifestations including edema and frothy urine. Renal biopsy findings confirmed a diagnosis of MCD. This presentation aligns with reports that 5% to 10% of MCD cases may manifest without overt edema, particularly in autoimmune contexts.^[1]^

Though dynamic imaging of salivary glands (nuclear medicine imaging) showed that the uptake, secretion and excretion of the glands were generally normal, there have been some reported cases of SS with MCD,^[7]^ which studies have indicated that TIN usually occurs before sicca symptoms and increases the possibility of worsening renal function.^[8]^ Given the patient’s history of persistent hypokalemia and alkaline urine, combined with the strongly positive indicators of anti-SSA/Ro52 (+++) and anti-SSB (++), highly suspected SS. However, a salivary gland biopsy (the gold standard for SS diagnosis) was not performed. According to the kidney pathology and the Sjögren syndrome related kidney injury standard reported in the literature,^[9]^ she was diagnosed as MCD and acute TIN or (Sjögren syndrome kidney injury).

She was treated with intravenous gamma globulin 20 g daily for 5 consecutive days, pulse methylprednisolone for 3 days then switched to prednisone 50 mg daily. Hydroxychloroquine 100 mg daily and cyclophosphamide 0.4 g daily was also administered to suppress the immune response. The patient responded to this therapy well and her condition gradually improved, as shown in her serial laboratory findings. After 14 days, the patient’s nephrotic syndrome symptoms were relieved. After 18 days, the patient’s muscle strength improved and she was discharged from the hospital (presented timeline of treatment course in Table 1). During a 24 months follow-up, she did not experience renal or neurological symptoms or disease recurrence.

**Table 1:** Timeline of immunotherapy and clinical course.

Hospital day	Treatment	Symptoms and laboratory findings	Outcome
Day 9	• Methylprednisolone 250 mg IV qd • Hydroxychloroquine 100 mg PO bid • IVIg 20 g IV qd initiation • Enoxaparin anticoagulation	• Syncopal episode (previous day) • Lower limb muscle strength: 2/5 (MRC scale) • Absence of edema • Nephrotic-range proteinuria (6.544 g/24 h) (Day 1) • Albumin: 30.6 g/L (Day 1) • ANA (+, 1:1000), anti-SSA/Ro52 (+++), anti-SSB (++), p-ANCA (+), and AMA-M2 (+) (Day 1) • Hypokalemia (K⁺: 2.87 mmol/L) • Severe hypoalbuminemia (Alb: 20.4 g/L)	Diagnosed with MCD, strongly suspected SS. Initiation of immunomodulatory therapy
Day 10	• Methylprednisolone ↑ 500 mg IV qd • IVIg 20 g IV qd	• Improved muscle strength: 3/5 • Nephrotic-range proteinuria (9.50 g/24 h) • Albumin: 19.5 g/L • Elevated CSF protein (1.08 g/L) with albuminocytologic dissociation • Elevated transaminases (ALT: 63 U/L, AST: 56 U/L)	Confirmed diagnosis of Guillain–Barré syndrome. Neurological improvement.
Day 11–13	• Methylprednisolone 500 mg IV qd • IVIg 20 g IV qd • Cyclophosphamide 0.2 g IV (first dose)	• Muscle strength: 3⁻/5 • Proteinuria (1.632 g/24 h) • Persistent hypokalemia (K⁺: 2.95 mmol/L) • Profound hypoalbuminemia (Alb: 18.8 g/L)	Progressive neurological recovery. Incomplete electrolyte correction. Resolution of nephrotic syndrome.
Day 16	• Methylprednisolone ↓ 40 mg IV qd • Cyclophosphamide 0.2 g IV (second dose) • Hydroxychloroquine ↑ 200 mg PO bid	• Significant muscle strength improvement: 4⁻/5 • Markedly reduced proteinuria (0.135 g/24 h) • Albumin: 20.4 g/L • Hypergammaglobulinemia (γ-globulin: 50.9%)	Near-normalization of muscle strength. Resolution of nephrotic syndrome.
Day 18	Discharge medication: • Prednisone 50 mg PO qd • Hydroxychloroquine 200 mg PO bid	• Muscle strength: 4/5 • ANA (+, 1:320), anti-SSA/Ro52 (+++), anti-SSB (+) • Severe hypoalbuminemia (Alb: 20.4 g/L) • Absence of edema • Normal-range proteinuria	Discharge with neurorehabilitation plan

Alb = albumin, ALT = alanine aminotransferase, ANA = antinuclear antibodies, anti-SSB = anti-Sjögren syndrome antigen B, AST = aspartate aminotransferase, bid = twice daily, CSF = cerebrospinal fluid, IV = intravenous, IVIg = intravenous immunoglobulin, p-ANCA = perinuclear anti-neutrophil cytoplasmic antibodies, PO = per os, qd = once daily.

## 3. Discussion

We reported a young woman whose symptoms first manifested as rapidly progressive, ascending symmetric paralysis following infectious illness, with sensory numbness, peaking in severity within a few days. She also had massive proteinuria, hypoproteinemia, and persistent hypokalemia. Serological testing revealed multiple autoantibody specificities: anti-SSA/Ro52 (+++), anti-SSB (++), ANA (positive at 1:1000 titer), p-ANCA (+), and AMA-M2 (+). Cerebrospinal fluid examination showed protein–cell dissociation, and the electroneurogram showed demyelinating damage. At the same time, her renal biopsy showed MCD and TIN (SS related kidney injury). Based on the above analysis, we confirmed a diagnosis of GBS, MCD, with Sjögren syndrome kidney injury.

Literature review indicates an annual GBS incidence of 1 to 2 per 100,000 population.^[2]^ However, its concomitant occurrence with NS is exceptionally rare. To our knowledge, no comprehensive reports exist characterizing this comorbidity or quantifying its incidence. We have summarized representative published cases of NS-GBS co-occurrence in Table 2. To date, we have identified only 5 cases of MCD associated with GBS (1973–2022). Notably, in 80% (4 of 5) of these cases, neurological symptoms preceded nephrotic symptoms by 5 days to 3 weeks, and steroid therapy was effective in the same proportion of patients (Table 3). These shared characteristics closely resemble those observed in our case, suggesting a potential common immunopathogenic mechanism or pathological basis underlying both GBS and MCD.

**Table 2:** Representative reported cases of nephrotic syndrome (NS) associated with Guillan-Barre syndrome (GBS).

Year	Author	Age (years)/sex	Renal biopsy	Associated feature	Treatment	Outcome
1973 (first case)	Behan et al^[3]^	50/M	MN (IgG/IgM granular deposits, subepithelial electron-dense deposits)	Post-viral infection; cell-mediated hypersensitivity to nerve/kidney antigens; humoral antibodies to nerve/kidney	Prednisone (60 mg/day, 3 weeks) + 6-mercaptopurine (50–150 mg/day, 4 weeks)	Persistent proteinuria.
1982	Talamo and Borochovitz	63/M	MN (stage 1) + acute interstitial nephritis	Upper respiratory infection (3 weeks prior)	Prednisone 80 mg/day → tapered to 20 mg/day	Neurological improvement, nephrotic syndrome unresolved (no long-term follow-up)
1986	Murphy et al	69/M	MN	No recent infection; slightly elevated blood mercury (hair normal)	No immunosuppressive therapy used	Rapid neurological deterioration; respiratory failure requiring ventilation; died at 3 weeks (autopsy: MGN + demyelination)
		52/M	MN	Upper respiratory infection history; hypertension; renal calculus	Prednisolone 60 mg/day → tapered	Slow neurological improvement; persistent nephrotic syndrome; sudden death at 6 months (autopsy: pulmonary embolism + renal vein thrombosis)
1998	Keven et al	57/M	MN	p-ANCA positivity	Not specified (diagnostic criteria for GBS confirmed)	Fatal outcome (details not fully specified)
2013	Filippone et al	69/M	MN	Negative IgG4 staining (suggesting secondary MN), severe axonal GBS variant	Intravenous immunoglobulin (IVIg) × 2 courses (discontinued due to AKI)	Fatal (refractory shock, quadriplegia, VT arrest)
1993	Careless et al	73/F	FSGS	Preceding viral URTI; proteinuria (2.6 g/day), hematuria	Plasmapheresis (partial response) → Prednisone (80 mg/day tapered)	Complete remission of GBS and NS after steroids
1998	Heckmann et al	46/M	FSGS	Not specified	No response to IVIg or plasmapheresis; remission with corticosteroids	Remission of GBS and NS
2007	Lim et al	22/M	FSGS collapsing variant	*Campylobacter jejuni* enteritis; Transient macroscopic hematuria	IV Immunoglobulin (IVIg)	Complete remission: neurological recovery; proteinuria 0.15 g/day at 16 months
2008	Souayah et al	49/M	FSGS	Relapsing GBS; hypertension (206/119 mm Hg)	IVIg (initial response, later failure) → Plasmapheresis + Prednisone (60 mg/day tapered)	Full recovery from GBS and NS; persistent low-dose steroids needed

Five reported cases of GBS combined with MCD are summarized in Table 3.

AKI = acute kidney injury, F = female, FSGS = focal segmental glomerulosclerosis, GBS = Guillain–Barré syndrome, IgG/IgM = immunoglobulin G/immunoglobulin M, IVIg = intravenous immunoglobulin, M = male, MCD = minimal change disease, MN = membranous nephropathy, NS = nephrotic syndrome, p-ANCA = perinuclear anti-neutrophil cytoplasmic antibodies, URTI = upper respiratory tract infection, VT = ventricular tachycardia.

**Table 3:** Reported cases of minimal change disease (MCD) associated with Guillan-Barre syndrome (GBS).

Year	Author	Age (years)/sex	Renal biopsy	Clinical feature	Associated feature	Treatment	Outcome
1980 (first case)	Frelich et al	44/M	Lipoid nephrosis	Neurological symptoms first appeared (paresthesia in hands/feet), NS detected within 1 week of hospitalization	T cell lymphocytopenia, lymphocytotoxic antibodies, depressed lymphocyte mitogenesis, and anergy	The immunological abnormalities resolved when the polyradiculoneuritis and lipoid nephrosis remitted. Immunosuppressive agents were not used	Improved after 8 months without immunosuppressive therapy
1996	Kitamura et al	34/M	MCD	Neurological symptoms first appeared (hand paresthesia), Proteinuria detected simultaneously with limb weakness/gait disturbance (~2 weeks later)	No immunological abnormalities noticed	Treated with prednisone, proteinuria and neurological status improved	Complete remission with prednisone, free of symptoms for 3 years
2002	Chen et al	55/F	Minimal change glomerulopathy with moderate tubulointerstitial nephritis	Limb weakness/numbness and foamy urine/edema appeared concurrently	Occupational exposure to an organic solvent, which contained acetone	Prednisone used and the occupational cleaning solution removed. The proteinuria decreased from 15.56 g to 4.99 g in 2 months	Partial remission, neurological improvement
2003	Ilyas et al	17/M	MCD	Neurological symptoms first appeared, proteinuria detected 5 days after GBS diagnosis	Streptococcus pneumoniae	Complete remission with prednisone over 18 months of follow-up	Complete remission with prednisone, proteinuria-free for 18 months
2007	Bouyahia	3/M	MCD	Neurological symptoms first appeared (limb weakness), NS developed 3 weeks after GBS onset	No immunological abnormalities noticed	Complete remission with prednisone over 24 months of follow-up	Complete remission with prednisone, proteinuria-free for 24 months
2019	Guo et al (present study)	31/F	Minimal change glomerulopathy with acute interstitial nephritis (or Sjögren syndrome kidney injury)	Neurological symptoms first appeared (fever/fatigue and limb numbness/weakness), Proteinuria was detected after admission.	B cell lymphocytopenia, positive for anti-SSA/SSB/RO52/RO60 Ab, positive for p-ANCA	Intravenous administration of gamma globulin, gradually decreasing glucocorticosteroids, cyclophosphamide immunosuppressants, No renal or neurological symptoms were observed over 24 months of follow-up	Complete remission and neurological improvement

Anti-SSA/SSB/Ro52/Ro60 Ab = anti-SSA: anti-Sjögren syndrome antigen A/SSB/Ro52/Ro60 antibodies, F = female, GBS = Guillain–Barré syndrome, IVIg = intravenous immunoglobulin, M = male, MCD = minimal change disease/lipoid nephrosis, NS = nephrotic syndrome, p-ANCA = perinuclear anti-neutrophil cytoplasmic antibodies, TIN = tubulointerstitial nephritis.

GBS is an inflammatory polyneuropathy mediated by autoantibodies and complements.^[2]^ Although the exact pathogenesis is unclear at present, it is generally considered to be mediated by humoral immunity and autoimmune responses, likely triggered by pathogen infection, molecular mimicry, resulting in production of anti-ganglioside antibodies, and complement activation.^[10]^ Molecular mimicry, the dual recognition of structures of a microbe and host, is the mechanism by which infections trigger cross-reactive antibodies or T cells leading to the symptoms of autoimmune disease. This phenomenon of molecular mimicry has been confirmed in GBS and overlaps with the mechanism of MCD.^[11]^

GBS is prone to cause NS secondary to immune disorders, especially in patients with autonomic dysfunction. Lymphocytes play a direct role in the pathogenesis of MCD, mainly causing podocyte damage.^[1]^ It has been hypothesized that this may be mediated by T cell lymphokines (proinflammatory T-helper cells, which produce interleukin-2, alpha-interferon, and T lymphocytotoxin, and CD8+ cytotoxic T cells).^[5]^ Disordered T cells release cytokines or infiltration factors, which injures podocyte foot processes. These specific autoantibodies are thought to arise from molecular mimicry of previous infections, and cross-react with 1 or more antigens expressed on the podocyte membrane. In our case, the presence of IgG‐, IgM+ in immunofluorescence, which may be podocytopathy caused by the immune response to the common antigen of peripheral nerves and glomeruli.^[11]^ Interestingly, Sjögren syndrome with renal involvement is associated with circulating immune complexes. The patient’s granular ANA pattern and robust positivity for anti-SSA/Ro/SSB antibodies reinforced SS diagnosis, while p-ANCA suggested a potential vasculitic component. These autoantibodies collectively indicate molecular mimicry-driven B/T-cell dysregulation, linking GBS, MCD, and SS.^[12]^ This is not a coincidence.

Anti-neutrophil cytoplasmic antibodies (ANCA) are antibodies that target proteins in the granules of neutrophils and lysosomes of monocytes. Patients with ANCA positivity are most commonly seen with primary renal involvement (especially p-ANCA), though the lesion type is rarely seen as microscopic lesions^[13]^, K Keven study found the same cases as ours (MCD with p-ANCA positive).^[14]^ ANAs have been shown to be prognostic indicators for rheumatic immune diseases affecting the kidneys. However, they are only detected in the blood and not in the kidney tissue, which does not give us much insight for now. But the elevation of ANCA and ANAs titers is highly likely to cause renal disease recurrence, which titer is reduced by treatment with cyclophosphamide and glucocorticoids.

In GBS-associated NS, renal injury is self-limited and resolves as neuropathy improves.^[11]^ In our case, however, the patient’s renal disease appeared to be persistent and at risk of worsening, for which we initiated clinical intervention. In previously reported cases treating GBS with MCD, prednisone and cyclophosphamide therapy resulted in relief of proteinuria and neurological symptoms. Meanwhile, intravenous immunoglobulin is a recognized immunotherapy agent for accelerating GBS recovery and alleviating neuropathy. In multiple cases of GBS and NS complications, it was indicated that the combination of intravenous immunoglobulin, prednisone and cyclophosphamide could basically restore muscle strength and kidney symptoms.^[15]^ In cases of SS complicated by MCD, hydroxychloroquine (a first-line SS therapy) enhances the efficacy of glucocorticoids in alleviating renal manifestations and significantly contributes to overall disease control.^[12]^ The triple immunosuppressive regimen combining glucocorticoids, cyclophosphamide, and hydroxychloroquine targets complementary immune pathways.

## 4. Conclusion

This is a case report of a rare concurrent GBS, MCD, and Sjögren syndrome kidney injury in a single patient. GBS mediated autoimmune disorders may precede the occurrence of MCD, suggesting that molecular mimicry and ANCA positivity may induce glomerular inflammation, alter or expose isolated epitopes in GBM, and subsequently trigger the production of antibodies in renal tissue. A significant proportion of GBS also present with autoimmune-mediated, circulating ANA or p-ANCA, suggesting that the co-existence of these 2 rare diseases in a single patient is no accident. In addition, considering that neurological symptoms often present initially, it is important to be alert to the potential development of renal disease later. Routine urinalysis and quantitative proteinuria assessment should be implemented for all GBS patients. This is particularly crucial when clinical features such as edema or unexplained hypoalbuminemia emerge. Early detection can mitigate irreversible nephron damage. Patients with too many combined factors are at risk for progression or recurrence of kidney disease, so we have a breakthrough in the treatment options. In order to effectively prevent or slow down the above conditions, induction and maintenance of immunosuppression are considered. But from a nephrological standpoint, the optimal approach to management in such cases is unknown.

## Author contributions

**Writing – original draft:** Ping Guo.

**Writing – review & editing:** Ping Guo, Ping Zhang, Jia Wei Zhao, Amanda Y. Wang, Wei Wang.
